# Optimal Conservative Therapy Use among Adult Cure Glomerulonephropathy Participants with IgA Nephropathy

**DOI:** 10.34067/KID.0000000000000306

**Published:** 2023-11-14

**Authors:** Arun Rajasekaran, Maria Larkina, Bruce A. Julian, Pietro A. Canetta, Bethany A. Roehm, Myda Khalid, Laura H. Mariani, Dana V. Rizk

**Affiliations:** 1 Division of Nephrology, University of Alabama at Birmingham, Birmingham, Alabama; 2 Division of Nephrology, University of Michigan, Ann Arbor, Michigan; 3 Division of Nephrology, Columbia University Irving Medical Center, New York, New York; 4 Division of Nephrology, University of Texas Southwestern Medical Center, Dallas, Texas; 5 Division of Pediatric Nephrology, Indiana University, Indianapolis, Indiana

**Keywords:** ACE inhibitors, IgA, IgA deposition, IgA nephropathy, immunosuppression, renin angiotensin system

## Abstract

- Optimal supportive therapy with BP and proteinuria control is pivotal in treating patients with IgA nephropathy.
- Suboptimal treatment of hypertension and proteinuria persisted in many patients with IgA nephropathy in the Cure Glomerulonephropathy Network study.
- Many patients had above-target proteinuria despite optimal BP control and may benefit from novel therapies or clinical trials.

## Introduction

IgA nephropathy is the most common primary glomerulonephritis, affecting approximately 2.5 per 100,000 persons worldwide, and is a leading cause of CKD. Up to 40% of patients with IgA nephropathy progress to kidney failure within 20 years of diagnostic biopsy.^1^ Furthermore, a diagnosis of IgA nephropathy reduces the expected life span by about a decade. Importantly, this increased mortality risk seems to be mediated by the occurrence of kidney failure, suggesting that measures to slow disease progression may mitigate this mortality risk.^2^ Adverse patient outcomes, including initiation of dialysis or death, have been correlated with the presence of clinical risk factors at the time of biopsy diagnosis, including proteinuria >1 g/d, sustained hypertension, and severity of pathologic findings on the basis of the revised Oxford classification—a structured and reproducible scoring system for evaluating the light microscopy features of IgA nephropathy.^3^ Clinical outcomes significantly improve if proteinuria ≤1 g/d is maintained with therapeutic interventions^4^; thus, reduction of proteinuria has been accepted as a surrogate end point to assess the efficacy of treatment in delaying disease progression.^4,5^ The Kidney Disease: Improving Global Outcomes (KDIGO) guidelines, published in 2012 and updated in 2021, emphasized robust supportive measures in patients with proteinuric IgA nephropathy at high risk of disease progression. Recommended interventions include optimal BP control; preferential use of maximal tolerated or allowed doses of a renin-angiotensin-aldosterone system inhibitor (RAASI); addressing cardiovascular risk, including hyperlipidemia; and adopting a healthy lifestyle. For patients with persistent proteinuria >1 g/d, the 2021 guidelines recommend consideration of clinical trials.^6^ In line with these recommendations, all ongoing clinical trials require these conservative measures to be in place before assessing patient eligibility.

We sought to ascertain the prevalence of the recommended robust conservative therapy at enrollment and longitudinally across 6 years after the initial kidney biopsy among well-characterized adults with IgA nephropathy in the National Institutes of Health–sponsored Cure Glomerulonephropathy Network (CureGN) study. We anticipated that the findings would identify the proportion of adults in this population who would benefit from more intensive conservative treatment and consideration for clinical trial enrollment.

## Methods

CureGN (https://curegn.org/) is a National Institutes of Health–funded, longitudinal, prospective, observational study of patients with four glomerular diseases. Since its inception, 2718 adult and pediatric patients with biopsy-proven minimal change disease, focal segmental glomerulosclerosis, membranous nephropathy, and IgA nephropathy (including IgA vasculitis with nephritis) have been enrolled across 66 centers. Patients with diabetes before biopsy were excluded. Eligible first-time diagnostic biopsies had been performed within 5 years of study enrollment. After the enrollment visit, study visits occurred within contiguous 4–6-month intervals throughout the study period wherein BP and other clinical parameters were recorded and biospecimens collected. Enrollment and subsequent yearly visits were in person while other visits were conducted in person or remotely (telephone or email). During in-person visits, a focused physical examination with measurement of BP was performed and patient-reported outcome measures were assessed, including medication adherence and clinical symptoms.^7^ BP was ascertained as follows: Participants were seated for approximately 5 minutes, followed by two seated BP measurements. The second seated BP reading was recorded.

Our study included only adults (18 years or older at the time of initial biopsy) with biopsy-proven IgA nephropathy. Patients with IgA vasculitis with nephritis were excluded. Per CureGN protocol criteria, participants were categorized as having incident disease (IgA nephropathy diagnosis by kidney biopsy within 6 months of CureGN enrollment) or prevalent disease (IgA nephropathy diagnosis ≥6 months before CureGN enrollment). Demographics and clinical data, including BP measurements and medications, were ascertained at enrollment and each subsequent annual in-person visit. The use of RAASIs, including angiotensin-converting enzyme inhibitors (ACEis), angiotensin II receptor blockers (ARBs), and mineralocorticoid receptor antagonists (MRAs), was documented at each visit. Maximal permissible dose was based on the manufacturer's maximal recommended dose in the package inserts. Patients with IgA nephropathy were considered to be on maximal tolerated RAASI dosage when RAASIs, including ACEis/ARBs/MRAs, were (*1*) used at the maximal permissible dose as monotherapy or combination therapy with ACEis/ARBs and MRAs, regardless of the BP reading during the visit; (*2*) used up to the highest permissible dose as monotherapy or combination therapy with ACEis/ARBs and MRAs, with antihypertension medication(s) of another class and BP <125/75 mm Hg; or (*3*) used at a dose up to the highest permissible dose as monotherapy or combination therapy, with ACEis/ARBs and MRAs with serum potassium level ≥5.2 mmol/L during the study visit. Locally collected laboratory data performed for routine clinical care, including urine protein-to-creatinine ratio (UPCR), serum potassium level, eGFR calculated with the 2021 CKD Epidemiology Collaboration creatinine-based formula,^8^ and serum total cholesterol level, were recorded at each visit or on the date closest to that visit at an interval of ±60 days.

Treatment goals were based on the 2012 KDIGO guidelines for the management of patients with glomerular diseases available at CureGN study inception (December 2014). KDIGO recommendations included long-term treatment with RAASIs (preferentially ACEi or ARBs) for proteinuria ≥1 g/d, with up-titration of dosage as tolerated to achieve proteinuria <1 g/d. BP treatment goals were <130/80 mm Hg for patients with <1 g/d proteinuria and <125/75 mm Hg when initial proteinuria was ≥1 g/d.^9^ In this study, patients with IgA nephropathy were considered to have implementation of adequate conservative therapy if they were on a maximal permissible or tolerated dose of a RAASI as monotherapy or combination therapy with alternate antihypertensive class medication(s), with BP <130/80 mm Hg and UPCR <1 g/g or BP <125/75 mm Hg and UPCR ≥1 g/g. Hypercholesterolemia was defined as serum total cholesterol >200 mg/dl or being on a cholesterol-lowering medication that entails statin monotherapy or combination therapy of a statin and ezetimibe.

Descriptive statistics were used to characterize the cohort at the enrollment visit and at cross-sections spanning time points up to 6 years from the initial diagnostic kidney biopsy. All patients could contribute to cross-sectional study data for more than 1 year if they continued to follow through with the CureGN study during continuous years. The proportion of patients using maximally tolerated RAASIs was described overall and by incident and prevalent participants. The proportions of patients achieving BP and proteinuria targets at time points after biopsy were also described.

## Results

**Figure 1:**
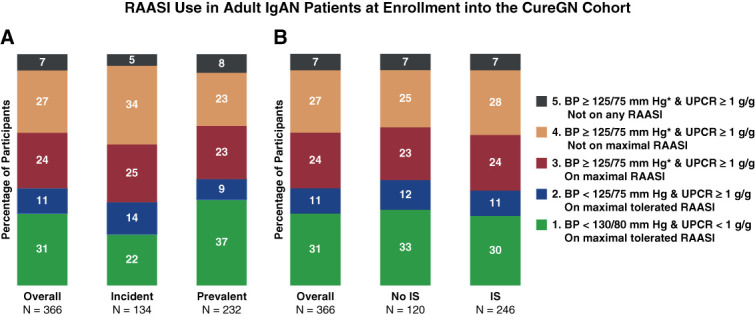
**RAASI use in adult patients with IgA nephropathy at enrollment into the CureGN cohort.** (A) RAASI use and optimal conservative treatment among adult incident and prevalent patients with IgA nephropathy at CureGN enrollment. (B) RAASI use and optimal conservative treatment among adult patients with IgA nephropathy not on immunosuppression medication and on immunosuppression medication at CureGN enrollment. Incident: per CureGN protocol, participants enrolled within 6 months of kidney biopsy; otherwise, participants were in the prevalent group; total *N*=366 with reported BP and UPCR at the enrollment visit. Usual maximum RAASI dose was based on manufacturer's recommendations. *Categories are mutually exclusive, wherein categories 3/4/5 encompass participants with BP ≥125/75 mm Hg and UPCR ≥1 g/g and BP ≥130/80 mm Hg and UPCR <1 g/g. The red, orange, and yellow bars reflect patients who are not meeting 2012 KDIGO guidelines for optimal conservative management. Blue and green bars reflect patients who are receiving optimal management per 2012 KDIGO guidelines. RAASI, renin-angiotensin-aldosterone system inhibitor; CureGN, Cure Glomerulonephropathy Network; KDIGO, Kidney Disease: Improving Global Outcomes; UPCR, urine protein-to-creatinine ratio; IS, immunosuppressant.

Patient characteristics at enrollment into the CureGN study are presented in Table 1. Five hundred two adult patients with IgA nephropathy (165 incident and 337 prevalent) were included. Three hundred sixty-six patients (73%) had BP and proteinuria data available at enrollment. The mean age was 40 years, mean eGFR was 65 ml/min per 1.73 m^2^, and mean UPCR was 1.7 g/g; incident patients had higher proteinuria than prevalent patients (UPCR, 2.4 versus 1.3 g/g). Hypercholesterolemia was present in 58% of the patients, with 48% on pharmacotherapy. RAASI was in use for 71% of the cohort, although only 41% were on a maximal tolerated dose. Immunosuppressant (IS) was used by 33% of the cohort, higher in incident (48%) than prevalent (26%) patients. At enrollment (median 399 days from initial biopsy), only 42% of the patients had optimal BP control for degree of proteinuria per the 2012 KDIGO guidelines.^9^ Of the 58% patients with suboptimal BP and proteinuria, 7% were not on RAASIs, 27% were on submaximal tolerated RAASIs, and 24% were on maximal tolerated RAASIs, but with BP not at goal. At enrollment, only 36% of incident patients and, surprisingly, just 46% prevalent patients had attained optimal supportive care. Among patients not taking an IS at enrollment, 55% had suboptimal BP and proteinuria, reflecting suboptimal conservative treatment (Figure 1).

**Table 1:** Descriptive characteristics of adult patients with IgA nephropathy at enrollment visit into the Cure Glomerulonephropathy Network

Characteristic	Overall	Incident	Prevalent
Participants, *N*	502	165	337
Years since biopsy (minimum, maximum)	1.7 (0, 6)	0.2 (0, 0.5)	2.4 (0.5, 6)
Sex: male, % (*n*)	58 (290)	68 (113)	53 (177)
Race: White, % (*n*)	75 (378)	80 (132)	73 (246)
Race: Black, % (*n*)	5 (24)	3 (5)	6 (19)
Race: Asian, % (*N*)	16 (79)	15 (24)	16 (55)
Age, yr (minimum, maximum)	40 (18, 81)	42 (18, 76)	39 (18, 81)
UPCR, g/g	1.7 (0.3, 1.9)	2.4 (0.6, 2.5)	1.3 (0.2, 1.6)
Serum potassium, mmol/L	4.4 (4.1, 4.7)	4.4 (4.0, 4.7)	4.5 (4.1, 4.8)
eGFR, ml/min per 1.73 m^2^	65 (40, 92)	65 (41, 94)	65 (40, 90)
BMI, kg/m^2^	29 (24, 32)	29 (24, 33)	28 (24, 32)
Systolic BP, mm Hg	124 (113, 135)	127 (115, 138)	123 (112, 131)
Diastolic BP, mm Hg	78 (70, 85)	78 (71, 85)	78 (70, 85)
On RAASI at visit, % (*n*)	71 (356)	72 (119)	70 (237)
On maximal dose of RAASI at visit, % (*n*)	41 (179)	39 (60)	42 (119)
On IS at visit, % (*N*)	33 (166)	48 (79)	26 (87)
Serum total cholesterol, mg/dl^a^	200 (170, 228)	212 (177, 255)	194 (167, 216)
Serum total cholesterol >200 mg/dl^a^, % (*n*)	42 (66)	52 (28)	37 (38)
On cholesterol-lowering class of medication, % (*n*)	48 (243)	50 (83)	47 (160)
Hypercholesterolemia^b^, % (*N*)	58 (292)	62 (103)	56 (189)
**BP control^c^, % (*n*)**			
1. BP <130/80 mm Hg and UPCR <1.0 g/g	23 (114)	18 (29)	25 (85)
2. BP <125/75 mm Hg and UPCR ≥1.0 g/g	8 (41)	12 (19)	7 (22)
3. BP ≥125/75 mm Hg^d^, on maximal RAASI	17 (87)	21 (34)	16 (53)
4. BP ≥125/75 mm Hg^d^, not on maximal RAASI	20 (99)	28 (46)	16 (53)
5. BP ≥125/75 mm Hg^d^, not on any RAASI	5 (25)	4 (6)	6 (19)
Missing BP data, % (*n*)	8 (39)	6 (10)	9 (29)
Missing UPCR data, % (*n*)	19 (97)	13 (21)	23 (76)

Data presented as % (*n*), mean (25%, 75% percentile), or mean (minimum, maximum) unless otherwise noted. BMI, body mass index; CureGN, Cure Glomerulonephropathy Network; eGFR, eGFR (calculated using 2021 CKD Epidemiology Collaboration [CKD-EPI] creatinine-based formula^8^); IS, immunosuppressant; RAASI, renin-angiotensin-aldosterone system inhibitor; UPCR, urine protein-to-creatinine ratio.

a Cholesterol data were available for only a limited number of participants.

b Hypercholesterolemia is defined as serum total cholesterol >200 mg/dl or being on a cholesterol-lowering medication that entails statin monotherapy or combination therapy of a statin and ezetimibe.

c At enrollment, 366 among 502 overall adult patients with IgA nephropathy had BP and proteinuria data available.

d Categories are mutually exclusive, wherein categories 3/4/5 encompass participants with BP ≥125/75 mm Hg and urine protein-to-creatinine ratio ≥1.0 g/g and BP ≥130/80 mm Hg and urine protein-to-creatinine ratio <1.0 g/g.

**Figure 2:**
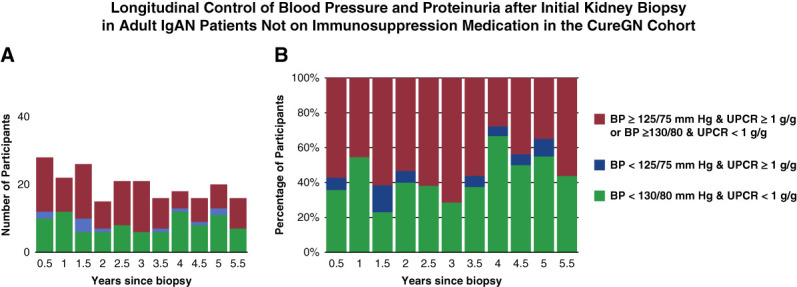
**Longitudinal control of BP and proteinuria after initial kidney biopsy in adult patients with IgA nephropathy not on immunosuppression medication in the CureGN cohort.** This figure shows the extent of the longitudinal control of BP and proteinuria for the adult patients with IgA nephropathy not on immunosuppression medication up to 6 years after the diagnostic biopsy who had a CureGN visit with non-missing BP and UPCR data in any of the 6-month intervals. (A) displays the data as numbers, and (B) displays the data as percentages. Initial kidney biopsy was at 0 years. Each patient contributed no more than 1 record per column, but some patients contributed to as many columns because they had non-missing BP and UPCR data or contributed to just a few intervals if they had fewer visits with complete data. The green and blue categories are considered patients who are receiving optimal management per 2012 KDIGO guidelines; red category patients are those with elevated BP and proteinuria (BP ≥125/75 mm Hg and UPCR ≥1 g/g or BP ≥130/80 mm Hg and UPCR <1 g/g) and are considered not optimized on the basis of the contemporaneous 2012 KDIGO guidelines available at the start of this study.

Table 2 summarizes longitudinal clinical data yearly over 6 years from the initial biopsy. We selected in-person visits as a priority per cross-section, followed by any other type of visit within ±6 months of patient's annual anniversary of the biopsy date. Using this metric for 502 patients, 480 patients (96%) had BP and proteinuria data available. RAASIs (including submaximal tolerated doses) were used in 68%–78% of these patients. Among patients who were never on an IS throughout this study, 28%–71% had suboptimal BP and proteinuria up to 6 years after biopsy (Figure 2). Significant percentages of patients (29%–38%) had persistence of BP ≥125/75 mm Hg and UPCR ≥1.0 g/g or BP ≥130/80 mm Hg and UPCR <1.0 g/g during the study period regardless of IS use. In addition, 23%–33% of the full cohort were on an IS and 60%–67% had hypercholesterolemia during this study (Table 2).

**Table 2:** Longitudinal characteristics of adult patients with IgA nephropathy in the Cure Glomerulonephropathy Network

Characteristic	Year 1	Year 2	Year 3	Year 4	Year 5	Year 6
Participants, *N*	253	264	251	235	204	157
Years since biopsy (minimum, maximum)	1 (0.5, 1.5)	2 (1.5, 2.5)	3 (2.5, 3.5)	4 (3.5, 4.5)	5 (4.5, 5.5)	6 (5.5, 6.5)
Age, yr (minimum, maximum)	41 (18, 76)	41 (18, 77)	43 (18, 78)	44 (18, 79)	45 (18, 81)	45 (20, 76)
UPCR, g/g	1.3 (0.2, 1.2)	1.2 (0.2, 1.4)	1.2 (0.2, 1.3)	0.9 (0.1, 1.1)	1.2 (0.2, 1.4)	1.3 (0.2, 1.7)
Serum potassium, mmol/L	4.5 (4.1, 4.7)	4.5 (4.1, 4.7)	4.5 (4.2, 4.8)	4.5 (4.2, 4.8)	4.5 (4.1, 4.8)	4.5 (4.1, 4.8)
eGFR, ml/min per 1.73 m^2^	66 (39, 94)	63 (35, 88)	64 (36, 89)	62 (38, 85)	57 (31, 84)	60 (33, 86)
Systolic BP, mm Hg	126 (114, 136)	124 (113, 133)	124 (113, 133)	124 (112, 134)	125 (115, 134)	125 (116, 135)
Diastolic BP, mm Hg	79 (71, 85)	77 (70, 84)	78 (70, 85)	78 (70, 86)	77 (70, 85)	79 (72, 84)
On RAASI at visit, % (*n*)	78% (198)	70% (186)	73% (183)	68% (159)	73% (149)	71% (112)
On IS at visit, % (*n*)	33% (84)	27% (71)	24% (61)	23% (55)	23% (47)	27% (43)
Serum total cholesterol, mg/dl^a^	187 (163, 210)	193 (166, 221)	182 (152, 206)	194 (158, 215)	195 (164, 224)	193 (159, 220)
Serum total cholesterol >200 mg/dl^a^, % (*n*)	34% (29)	38% (30)	32% (24)	36% (24)	40% (26)	37% (19)
On cholesterol-lowering class of medication, % (*n*)	58% (148)	59% (157)	61% (153)	54% (128)	57% (116)	59% (92)
Hypercholesterolemia^b^, % (*n*)	67% (169)	67% (176)	67% (168)	60% (141)	66% (134)	66% (104)
**BP category distribution, % (*n*)**						
1. BP <130/80 and UPCR <1.0 g/g	24% (60)	26% (68)	20% (50)	22% (51)	21% (42)	11% (18)
2. BP <125/75 and UPCR ≥1.0 g/g	6% (14)	3% (7)	2% (4)	4% (10)	5% (11)	3% (5)
3. BP ≥125/75 and UPCR ≥1.0 g/g or BP ≥130/80 and UPCR <1.0 g/g	38% (95)	33% (87)	36% (90)	31% (73)	33% (67)	29% (46)
Missing BP data, % (*n*)	7% (17)	8% (22)	8% (21)	7% (17)	5% (11)	6% (10)
Missing UPCR data, % (*n*)	26% (67)	30% (80)	34% (86)	36% (84)	36% (73)	50% (78)

Table presents % (*N*), mean (25%, 75% percentile), or mean (minimum, maximum) unless otherwise noted. Only 480 among 502 adult patients with IgA nephropathy had BP and proteinuria data available for in-person visits selected as priority per cross-section, followed by any other type of visit within ±6 months of patient's annual anniversary of the biopsy date. CureGN, Cure Glomerulonephropathy Network; eGFR, eGFR (calculated using 2021 CKD Epidemiology Collaboration [CKD-EPI] creatinine-based formula^8^); IS, immunosuppressant; RAASI, renin-angiotensin-aldosterone system inhibitor; UPCR, urine protein-to-creatinine ratio.

a Cholesterol data were available for only a limited number of participants.

b Hypercholesterolemia is defined as serum total cholesterol >200 mg/dl or being on a cholesterol-lowering medication that entails statin monotherapy or combination therapy of a statin and ezetimibe.

## Discussion

IgA nephropathy predominantly affects young patients with peak incidence in the second and third decades of life. The diagnosis imparts significant morbidity and is associated with increased mortality, mediated primarily by the development of kidney failure. Studies have shown that optimal supportive care modifies risk factors, potentially reducing the need for aggressive immunosuppressive therapies and delaying kidney function decline. In a study of 96 Indian patients with IgA nephropathy, remission (complete remission [CR] defined as proteinuria <0.5 g/d; partial remission defined as proteinuria <1 g/d with ≥50% reduction from baseline with ≤25% eGFR loss) was observed in 36.5% (CR, 6.3%) at 3 months and in 55.2% (CR, 31.3%) at 6 months with optimal ACEi/ARB use (72% patients received ≥75% of the maximal dose).^10^ The Supportive versus Immunosuppressive Therapy for the Treatment of Progressive IgA Nephropathy clinical trial included a 6-month run-in phase wherein conservative management (included maximal tolerated RAASIs with ACEis or ARBs to target BP <125/75 mm Hg and proteinuria <0.75 g/d, dietary counselling, and statin therapy) was optimized before randomization. After the run-in phase, 34% of enrolled patients were excluded from randomization as proteinuria declined to <0.75 g/d and no longer met the inclusion criterion.^11^

The 2012 KDIGO guidelines, with the updates in 2021, highlight the seminal role of BP control and proteinuria reduction using RAASIs coupled with hypercholesterolemia treatment and implementation of a healthy lifestyle. We sought to ascertain the implementation of these guidelines among a well-characterized cohort of patients enrolled in CureGN and followed longitudinally for 6 years after biopsy. Influence of optimal supportive care was studied in incident and prevalent patients separately at enrollment to assess whether conservative management had been optimized after diagnosis. Suboptimal BP and proteinuria control continued among incident and prevalent patients. Many participants did not have UPCR data available perhaps because UPCR testing was not performed in a consistent manner in these patients. Despite the well-established protective role of RAASIs in proteinuric CKD, including IgA nephropathy, 34% of participants at enrollment were not on maximal RAASI dosing despite their BP allowing higher dosing. Because enrollment was shortly (within 6 months) after diagnosis for incident patients, we also examined the data among prevalent patients; unfortunately, 31% remained on suboptimal RAASI treatment. In patients with data at the time of biopsy and yearly thereafter for 6 years, 58% at enrollment regardless of IS use and 28%–71% patients not on any IS during follow-up had suboptimal control of BP and proteinuria, with submaximal or no RAASIs. The latter may have been due to symptomatic hypotension or hyperkalemia; the observational nature of the study limits this inference.

The updated KDIGO guidelines released in 2021, almost 6 years after the CureGN study inception, were more stringent than the 2012 guidelines and further emphasized the importance of optimal supportive care, including RAASIs as much as tolerated/allowed. RAASI was recommended in the absence of hypertension if proteinuria was ≥0.5 g/d. If proteinuria persists above 0.75–1 g/d despite ≥90 days of optimal conservative management, there is a high risk of progressive loss of kidney function; the guidelines recommended that such patients participate in a therapeutic clinical trial, but they could be considered for a 6-month course of glucocorticoids.^6^ Therefore, application of the more stringent 2021 KDIGO guidelines to our cohort would likely show more patients at risk. These alarming results highlight the need to continue efforts to educate patients and care providers about the established merits of robust supportive care and demonstrate the mismatch between guideline-defined therapeutic goals and real-world achievement of those goals, even at centers of excellence.

We found that 32% of prevalent patients at study enrollment continued to have significant proteinuria despite maximal tolerated RAASIs or to have low-normal BP (≤125/75 mm Hg) for whom therapy cannot be intensified from a hemodynamic standpoint. In such patients, initiation of a sodium-glucose cotransporter-2 inhibitor represents another emerging avenue of supportive care in the management of IgA nephropathy. In recent trials, treatment with sodium-glucose cotransporter-2 inhibitors reduced the risk of progressive kidney disease, kidney failure, or death.^12,13^ This sizable subgroup of patients with suboptimal control could also qualify for other recently approved IgA nephropathy treatments and/or referral for disease-specific clinical trials.^14^

Our study has several limitations. Given the objective data capture, other pertinent subjective conservative measures for the management of IgA nephropathy were not assessed, including dietary limitations for sodium/protein consumption and smoking cessation. The CureGN cohort represents study participants being closely followed and monitored at major academic centers; thus, the findings may not be applicable to the general US population and international patients. Laboratory monitoring was performed per routine clinical practice, and testing had not been performed at some time points.

Nonetheless, the CureGN IgA nephropathy cohort is one of the largest well-characterized cohorts with prospective, longitudinal data. The granular medication data allowed ascertainment of RAASI and IS use and dosing. Our study highlights important findings about the current management of patients with IgA nephropathy and identifies opportunities for better, more holistic, management. In addition, a significant percentage of adult patients with IgA nephropathy remain at high risk of kidney disease progression despite optimal supportive therapy and may qualify for enrollment in clinical trials.

## Data Availability

All data are included in the manuscript and/or supporting information. Partial restrictions to the data and/or materials apply. Patient-level data cannot be shared due to the nature of the rare disease: Cure Glomerulopathy Network data are access-restricted and cannot be shared publicly. Data can be requested by investigators through the procedure for a CureGN ancillary study at www.curegn.org.
